# Intestinal Obstruction due to Colonic Lithobezoar: A Case Report and a Review of the Literature

**DOI:** 10.1155/2013/854975

**Published:** 2013-01-16

**Authors:** Metin Şenol, Zehra Ünal Özdemir, İbrahim Tayfun Şahiner, Hakan Özdemir

**Affiliations:** Department of General Surgery, Nevşehir State Hospital, 50000 Nevsehir, Turkey

## Abstract

Bezoar is defined as the accumulation of undigested foreign bodies or nutrients in the gastrointestinal tract. These foreign bodies can be hair (trichobezoar), fibers or seeds of vegetables and fruits (phytobezoar), or remnants of milk (lactobezoar) and stones (lithobezoar). Lithobezoar, the accumulation of stones in the digestive tract, is commonly seen in stomach. In this paper, a 7-year-old girl with colonic lithobezoar who presented with constipation, abdominal pain, and the history of pica was successfully treated by the extraction of the stones under general anesthesia.

## 1. Introduction

Colonic lithobezoar is a rare disorder and may cause mechanical intestinal obstruction in children [1]. It is mostly seen in patients with the history of iron deficiency anaemia and pica. Pica is an eating disorder typically defined as the persistent ingestion of nonnutritive substances [2]. We report a case of colonic lithobezoar in a child who was successfully evacuated under general anesthesia.

## 2. Case Report

A 7-year-old girl was admitted to the emergency room with abdominal pain and constipation. She had an absence of defecation for the past 3 days. In her medical history, she was treated for iron deficiency anaemia and she had the compulsive behavior of pica. 

Her vital parameters were normal and her general physical examination results were unremarkable. Abdominal examination revealed distention and mild tenderness with no signs of peritonitis. Irregular masses in the left lower quadrant of the abdomen were palpated. Rectal examination demonstrated hard masses like fecaloids. Some of these masses were extracted and they turned out to be stones along with stool.

**Figure 1:**
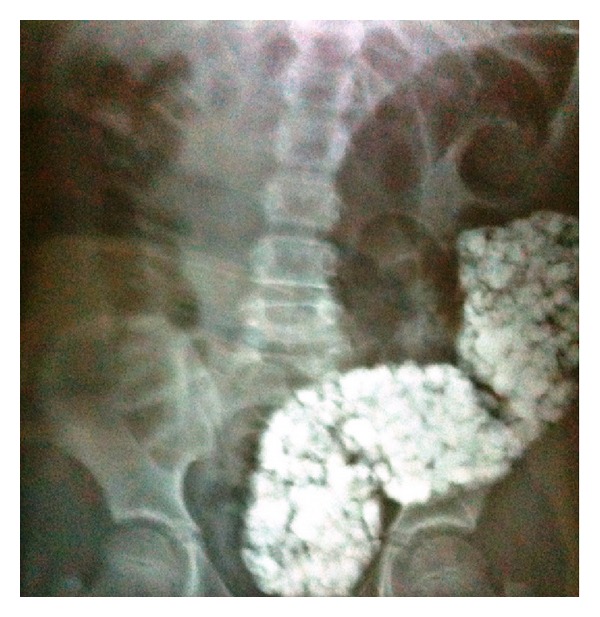
Abdominal X-ray.

The laboratory studies revealed hemaglobin 6.8 g/dL, hematocrit 22.5%, MCV 53.4 fL, ferritin 9.0 ng/mL, iron 14 mg/dL, and iron binding capacity 415 mg/dL, pointing out an iron deficiency anaemia. Abdominal X-ray demonstrated radioopaque masses of various sizes in the distal colon. There was no noticed air-fluid level (Figure 1). 

**Figure 2:**
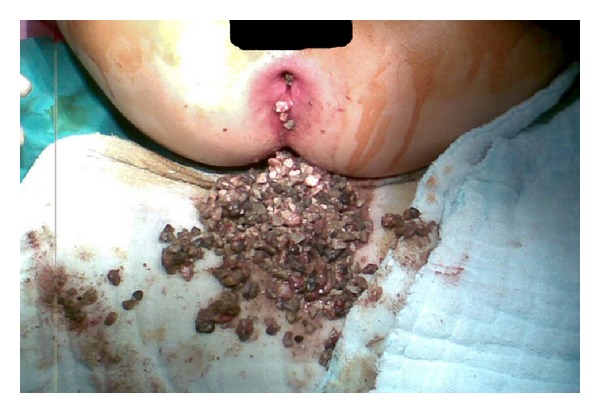
Anal dilatation and extraction of stones.

Along these results, the diagnosis of intestinal obstruction due to lithobezoar was made. Under general anesthesia, following anal dilatation, manual evacuation, and colonic lavage were done. Approximately 2 kilograms of stones in various sizes were extracted (Figure 2). She was followed up with abdominal X-ray and laboratory tests postoperatively. She continued to pass out stones till the postoperative second day. On the postoperative third day, her abdominal X-ray revealed no stones in the colon and no signs of colonic obstruction and perforation, thus she was discharged. Iron supplement was prescribed for her iron deficiency anaemia. She did not have a relapse in the followup for a year.

## 3. Discussion

Bezoar is the accumulation of undigested foreign bodies or nutrients in the gastrointestinal tract. These foreign bodies can be hair (trichobezoar), fibers or seeds of vegetables and fruits (pythobezoar), or remnants of milk (lactobezoar) and stones (lithobezoar) [1]. Although the stomach is the predilection site for bezoar, it can be rarely seen in colon and it may cause mechanical intestinal obstruction. Colonic lithobezoar in children is very rare, up until 2012, only 6 cases are reported [1, 3–7] (Table 1).

Colonic lithobezoar can be seen as a result of pica [3, 5]. Pica is characterized by persistent craving and compulsive eating of nonfood substances such as clay or soil, ice, and stone. The etiology of pica is multifactorial including iron deficiency anaemia, mental retardation, poverty, pregnancy, traditional eating habits, parental neglect, and low socioeconomic status. Pica has been reported to be associated with severe iron deficiency anaemia in up to half of patients; however, it is unclear whether pica causes or is the result of iron deficiency anaemia [2, 8, 9]. In this case, she had severe iron deficiency anaemia. Additionally she was the fifth child of her family with a low socioeconomic status. These can explain her compulsive behavior, pica. As her parents told, she sometimes eats soil and stone. At the time of this event, there was road construction in front of their house and she had swallowed the stones supposed to be used to build the road. 

Clinically patients present with signs and symptoms of colonic obstruction. In physical examination, abdominal distention and tenderness is mostly seen along with “colonic crunch sign” which is the palpation of the masses [6]. Stones can be palpated and extracted in rectal examination. A plain X-ray demonstrating various sized opacities is helpful; this sign is referred to as “corn on the cub” [6].

The treatment of colonic bezoars depends on the site of the colon and the type and the size of the substance [10]. In the literature, the previously reported cases of colonic lithobezoar were treated by anal dilatation and extraction of the stones under anesthesia. Surgery can be considered if this procedure fails or colonic injury occurs [1, 3–7]. In this case, we also performed manual evacuation of the colon under general anesthesia and she was treated successfully without surgery. 

In summary of the literature, colonic lithobezoar cases present with constipation, abdominal distension, and painful defecation. These children usually have no peritoneal signs but irregular mass at abdominal palpation and prickly mass at rectal examination. Most of them have pica with or without iron deficiency anaemia. Abdominal X-ray is suitable for diagnosis. Anal dilatation and extraction of the stones is enough for the treatment. 

## 4. Conclusion

Colonic lithobezoar is a rare disorder but it must be suspected in children with mechanical intestinal obstruction and the history of pica. It can be lethal by causing colonic obstruction moreover perforation, if it is undiagnosed. Evacuation of the colon by anal dilatation is usually enough and surgery is not required. Treatment of iron deficiency anaemia and pica could be the key for the prevention.

## Figures and Tables

**Table 1:** Colonic lithobezoar in children overview of literature.

Study	Age	Gender	Sign/Symptoms	Physical examination	Radiologic evaluation	Laboratory	Pica	Treatment
Narayanan et al. [3]	9	Male	C, AP	No peritoneal sign, prickly mass at rectal exam	X-ray	N/A	+	Laxative and rectal flushout
Numanoğlu and Tatli [1]	4	Male	C, abdominal distention	Irregular mass at palpation	X-ray	HCT:21, 1	N/A	Anal dilatation, extraction of stones
Mohammad [4]	8	Male	C, AP, hematochezia, bilious vomiting, abdominal distension	Moderate distension, PM no features of peritonitis	X-ray,	N/A	+	Anal dilatation, extraction of stones
Sheikh et al. [5]	9	Male	C, AP, failure to thrive, painful defecation	Moderate distension, PM, no features of peritonitis	X-ray	N/A	+	Anal dilatation, extraction of stones
Tokar et al. [6]	6	Female	C, AP	PM	X-ray	N/A	+	Anal dilatation, extraction of stones
Vijayambika [7]	6	Male	C, AP	Poorly localized tenderness	X-ray	N/A	+	Laxative and rectal flushout

C: constipation; AP: abdominal pain; PM: palpable mass.
